# Dandelion Extract Alleviated Lipopolysaccharide-Induced Oxidative Stress through the Nrf2 Pathway in Bovine Mammary Epithelial Cells

**DOI:** 10.3390/toxins12080496

**Published:** 2020-08-01

**Authors:** Yawang Sun, Yongjiang Wu, Zili Wang, Juncai Chen, You Yang, Guozhong Dong

**Affiliations:** College of Animal Science and Technology, Southwest University, Chongqing 400716, China; syaw507@swu.edu.cn (Y.S.); wuyongjiang@email.swu.edu.cn (Y.W.); wzl9698@swu.edu.cn (Z.W.); juncaichen@swu.edu.cn (J.C.); youy@swu.edu.cn (Y.Y.)

**Keywords:** dandelion extract, lipopolysaccharide, oxidative stress, Nrf2 pathway, bovine mammary epithelial cell

## Abstract

In practical dairy production, cows are frequently subjected to inflammatory diseases, such as high-grain diet-induced subacute ruminal acidosis (SARA) as well as mastitis and metritis. Under the circumstances, lipopolysaccharide (LPS) induces oxidative stress within the cow and in the mammary epithelial cells. It has implications in practical production to alleviate oxidative stress and to optimize the lactational function of the mammary epithelial cells. This study thus aimed to investigate the antioxidative effects of dandelion aqueous extract (DAE) on LPS-induced oxidative stress and the mechanism of DAE as an antioxidant to alleviate oxidative stress through the nuclear factor erythroid 2-related factor 2 (Nrf2) pathway in the bovine mammary epithelial cell line MAC-T cells. The cells were cultured for 48 h in six treatments including control (without LPS and DAE), LPS (100 ng/mL), DAE10 (100 ng/mL LPS and 10 μg/mL DAE), DAE50 (100 ng/mL LPS and 50 μg/mL DAE), DAE100 (100 ng/mL LPS and 100 μg/mL DAE), and DAE200 (100 ng/mL LPS and 200 μg/mL DAE), respectively. The results showed that cell viability was reduced by LPS, and the adverse effect of LPS was suppressed with the supplementation of DAE. Lipopolysaccharide-induced oxidative stress through enhancing reactive oxygen species (ROS) production, resulted in increases in oxidative damage marker concentrations, while 10 and 50 μg/mL DAE alleviated the LPS-induced oxidative stress via scavenging cellular ROS and improving antioxidant enzyme activity. The upregulation of antioxidative gene expression in DAE treatments was promoted through activating the Nrf2 signaling pathway, with DAE at a concentration of 50 μg/mL exhibiting the highest effect. Overall, DAE acted as an effective antioxidant to inhibit LPS-induced oxidative stress and as a potential inducer of the Nrf2 signaling pathway.

## 1. Introduction

Oxidative stress is a consequence of excessive reactive oxygen species (ROS) production, inadequate antioxidant potential, or a synchronization of both. The abundance of ROS under oxidative stress leads to the damage of cellular macromolecules, resulting in protein modification, lipid peroxidation and DNA damage, which further increases cell senescence, compromises cell function and threatens cell survival [1,2]. The excessive production of cellular ROS can be promoted by the inflammatory reaction.

In practical dairy production, cows are frequently subjected to inflammatory diseases, such as high-grain diet-induced subacute ruminal acidosis (SARA) as well as mastitis and metritis. During high-grain diet-induced SARA, the oxidative stress was triggered by lipopolysaccharide (LPS) in plasma and the liver, and the milk yield as well as milk composition were impaired in SARA cows [3]. Subclinical mastitis is a prevalent disease in dairy cows worldwide, which is mainly caused by the LPS released from invading pathogens, resulting in reduced milk production and large losses to the dairy industry. LPS is the cell wall component of Gram-negative bacteria, and it can elicit inflammatory response through binding with the Toll-like receptor 4 (TLR4). Then TLR4 initiates the activation of nuclear factor κB (NF-κB), which enhances the production of proinflammatory cytokines and leads to subsequent oxidative stress [4,5]. Intramammary administration with lipopolysaccharide (LPS) stimulated inflammatory response and caused subsequent oxidative damage to the mammary gland through the activation of transduction pathways that express proinflammatory cytokines [6].

As the main cell type in the udder, the primary function of mammary epithelial cells (MECs) is to synthesize milk protein, fat and other components. Moreover, MECs also act as the first line of defense in initiating an inflammatory response. Previous studies showed that the oxidative stress accompanying the inflammatory response negatively affects the cellular function of MECs and even leads to cell apoptosis [7]. Therefore, it has implications in practical dairy production to alleviate oxidative stress in order to optimize the lactational function of the MECs.

Dandelion (*Taraxacum officinale* (L.) Weber ex F.H. Wigg) is a perennial Compositae herb that is originally from Europe and broadly distributed in the Northern Hemisphere. Dandelion has a long history as a herbal remedy against disorders, such as digestive ailments and liver diseases [8]. The common biological activity of dandelion includes anti-inflammatory, anti-microbial, and antioxidative effects. In terms of antioxidant activity, researchers found the main bioactive compounds of dandelion are flavonoids and phenolic acids [9]. Concerning different antioxidant properties among various plant parts, previous studies used microsomal P450 systems as markers to detect the inhibitive efficiency of hydroxyl radical production in different dandelion extracts [10]. Both ethyl acetate and water extracts of dandelion flowers derived from ethanol extraction (70% *v*/*v*), which contain high concentrations of flavones luteolin, caffeic acids, and chlorogenic acids, were proved to have a strong inhibition of hydroxyl radical production and activity of radical scavenging [11]. The antioxidative abilities of dandelion extracts were demonstrated with improved activities of antioxidative enzymes and relieved oxidative damage under various stimuli-induced oxidative stresses [12,13,14]. Treatment with dandelion leaf and root extracts promoted endogenous antioxidative activities and improved the lipid profiles in cholesterol-fed rabbits [15], and similar results were also shown in rats or even in fish [16,17]. In consideration of the situation of dairy production and the global trend of promoting antibiotic-free husbandry, dandelion extract, with the potential of antioxidative and anti-inflammatory effects, could be a promising alternative to antibiotics in the dairy industry.

In response to intracellular oxidative stress, nuclear factor erythroid 2-related factor 2 (Nrf2) plays a crucial role as a regulator of intracellular redox status. Through interacting with the antioxidant response element (ARE), Nrf2 counteracts excessive ROS by activating the expression of genes encoding key antioxidant molecules and antioxidant enzymes [18]. Nrf2 is negatively mediated by the cytoplasmic repressor Kelch-like ECH-associated protein 1 (Keap1), which inhibits Nrf2 from translocating to the nucleus where it would interact with the ARE and induce gene transcription [19]. Recently, the Nrf2 pathway is highlighted in antioxidant research and targeting this signaling pathway has been proven to be an appropriate strategy to relieve oxidative stress [20].

Thus, the aim of this study was to investigate the antioxidative effects of dandelion aqueous extract (DAE) on LPS-induced oxidative stress and to explore the mechanism of DAE as an antioxidant to alleviate oxidative stress through the Nrf2 pathway in bovine MEC line (MAC-T) cells.

## 2. Results

### 2.1. Effects of LPS and DAE on Cell Viability and ROS Production

**Figure 1:**
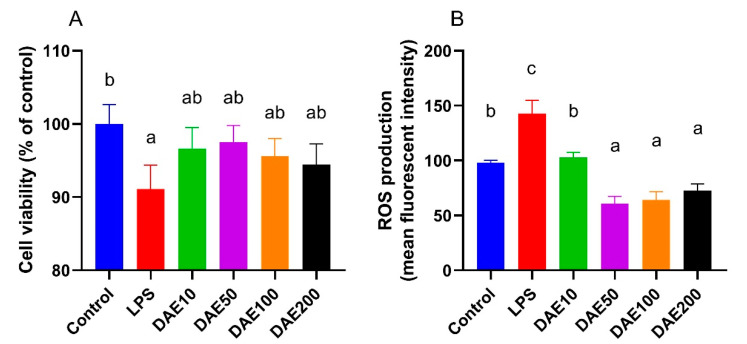
Effects of lipopolysaccharide (LPS) and dandelion aqueous extract (DAE) on cell viability (**A**) and cellular reactive oxygen species (ROS) production (**B**) in MAC-T cells. Columns without a common lowercase letter (a, b, c) indicate significant difference among treatments (*p* < 0.05). LPS: 100 ng/mL LPS; DAE10: 100 ng/mL LPS and 10 μg/mL DAE; DAE50: 100 ng/mL LPS and 50 μg/mL DAE; DAE100: 100 ng/mL LPS and 100 μg/mL DAE; DAE200: 100 ng/mL LPS and 200 μg/mL DAE.

As shown in Figure 1, LPS at the level of 100 ng/mL significantly decreased (*p* < 0.05) the cell viability of MAC-T cells, while the supplementation of DAE numerically improved the viability of MAC-T cells compared with the LPS treatment, although the difference did not attain a significant level. Cellular ROS production in the LPS treatment was significantly higher (*p* < 0.05) than in the control, and the addition of 10 μg/mL DAE effectively (*p* < 0.05) reduced LPS-induced ROS production. Moreover, MAC-T cells in the DAE50, DAE100, and DAE200 groups displayed significantly lower (*p* < 0.05) ROS production compared with the control.

### 2.2. Effects of LPS and DAE on the Concentration of Oxidative Damage Markers

**Figure 2:**
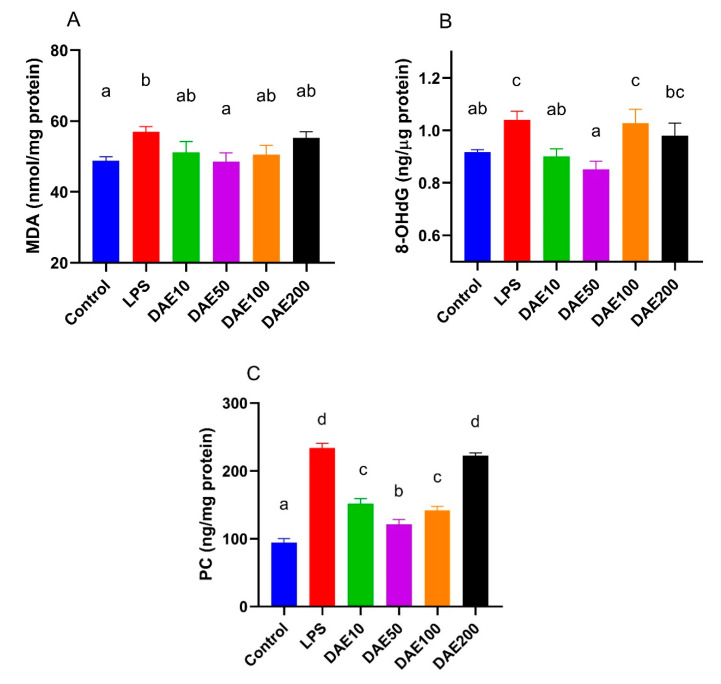
Effects of lipopolysaccharide (LPS) and dandelion aqueous extract (DAE) on the concentrations of oxidative damage markers, including malondialdehyde (MDA) (**A**), 8-hydroxy-2′-deoxyguanosine (8-OHdG) (**B**), and protein carbonyl (PC) (**C**) in MAC-T cells. Columns without a common lowercase letter (a, b, c, d) indicate significant difference among treatments (*p* < 0.05). LPS: 100 ng/mL LPS; DAE10: 100 ng/mL LPS and 10 μg/mL DAE; DAE50: 100 ng/mL LPS and 50 μg/mL DAE; DAE100: 100 ng/mL LPS and 100 μg/mL DAE; DAE200: 100 ng/mL LPS and 200 μg/mL DAE.

Under the treatment of 100 ng/mL LPS, cellular concentrations of all the oxidative damage markers increased significantly (*p* < 0.05) in comparison with the control. The addition of 50 μg/mL DAE significantly reduced (*p* < 0.05) malondialdehyde (MDA) concentration in cells, compared with the LPS group (Figure 2A). The supplementation of 10 or 50 μg/mL DAE significantly decreased (*p* < 0.05) cellular 8-hydroxy-2′-deoxyguanosine (8-OHdG) concentrations in contrast to the LPS treatment, while DAE levels of higher than 50 μg/mL did not significantly decrease 8-OHdG levels (Figure 2B) compared with the LPS group. Similar results were also observed in protein carbonyl (PC) concentration (Figure 2C); treatments with 10, 50, and 100 μg/mL DAE markedly reduced cellular levels (*p* < 0.05) of PC compared to LPS treatment, with the 50 μg/mL DAE displaying the best effect.

### 2.3. Effects of LPS and DAE on Antioxidant Enzyme Activity

**Figure 3:**
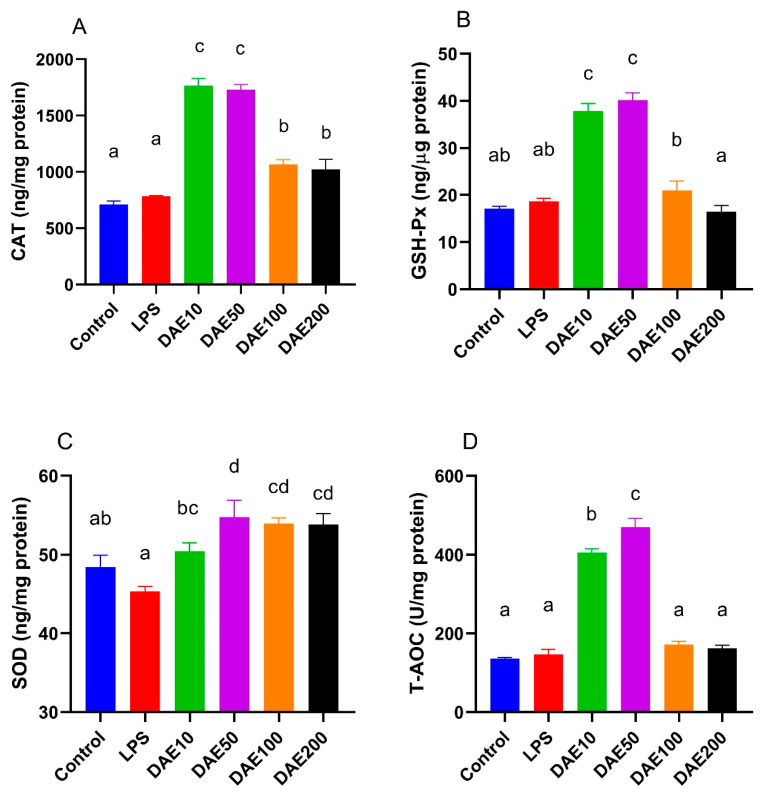
Effects of lipopolysaccharide (LPS) and dandelion aqueous extract (DAE) on the activities of antioxidants, including catalase (CAT) (**A**), glutathione peroxidase (GSH-Px) (**B**), superoxide dismutase (SOD) (**C**), and total antioxidant capacity (T-AOC) (**D**) in MAC-T cells. Columns without a common lowercase letter (a, b, c, d) indicate significant difference among treatments (*p* < 0.05). LPS: 100 ng/mL LPS; DAE10: 100 ng/mL LPS and 10 μg/mL DAE; DAE50: 100 ng/mL LPS and 50 μg/mL DAE; DAE100: 100 ng/mL LPS and 100 μg/mL DAE; DAE200: 100 ng/mL LPS and 200 μg/mL DAE.

The LPS did not affect the cellular activities of antioxidant enzymes; however, the supplementation of DAE improved antioxidant enzyme activity to various degrees (Figure 3). Activities of catalase (CAT), glutathione peroxidase (GSH-Px) and total antioxidant capacity (T-AOC) were significantly improved (*p* < 0.05) with the supplementation of 10 or 50 μg/mL DAE compared to the control and LPS groups. As regards superoxide dismutase (SOD) enzyme activities, cells with DAE supplementation had significantly higher (*p* < 0.05) activities than the LPS group. Cells in the DAE50, DAE100, and DAE200 groups displayed significantly higher (*p* < 0.05) SOD activities compared with the control, LPS, and DAE10 groups; and cells in the DAE100 and DAE200 groups exhibited significantly higher (*p* < 0.05) activities than the control and LPS groups.

### 2.4. Effects of LPS and DAE on the Protein Expression of Nrf2 and Keap1

**Figure 4:**
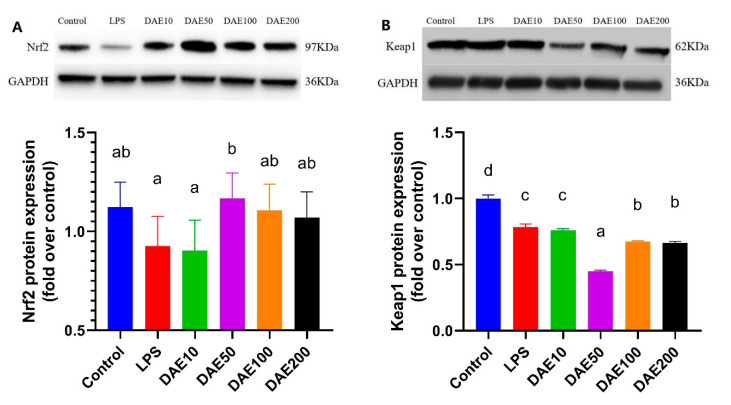
Effects of lipopolysaccharide (LPS) and dandelion aqueous extract (DAE) on the protein expression of nuclear factor erythroid 2-related factor 2 (Nrf2) (**A**) and kelch-like ECH-associated protein 1 (Keap1) (**B**) in MAC-T cells. Columns without a common lowercase letter (a, b, c, d) indicate significant difference among treatments (*p* < 0.05). GAPDH: glyceraldehyde-3-phosphate dehydrogenase; LPS: 100 ng/mL LPS; DAE10: 100 ng/mL LPS and 10 μg/mL DAE; DAE50: 100 ng/mL LPS and 50 μg/mL DAE; DAE100: 100 ng/mL LPS and 100 μg/mL DAE; DAE200: 100 ng/mL LPS and 200 μg/mL DAE.

As shown in Figure 4, the relative protein expression of Nrf2 was negatively affected by LPS treatment, while the supplementation of 50 μg/mL DAE significantly improved (*p* < 0.05) the protein expression of Nrf2. The expression of the Keap1 protein was significantly reduced (*p* < 0.05) under the treatment of LPS and DAE, and cells in the DAE50 groups exhibited significantly lower (*p* < 0.05) protein expression of Keap1 than in the control and other treatments.

### 2.5. Effects of LPS and DAE on the mRNA Expression of Antioxidative Genes

**Figure 5:**
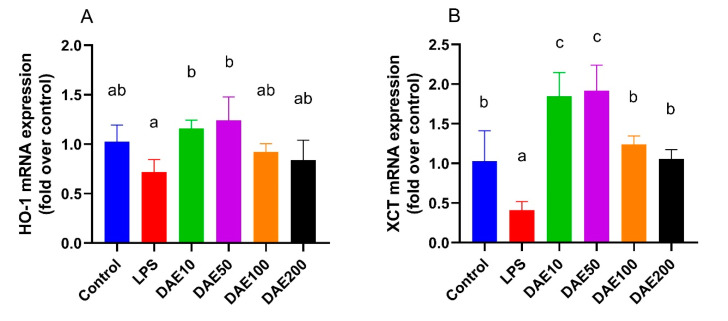

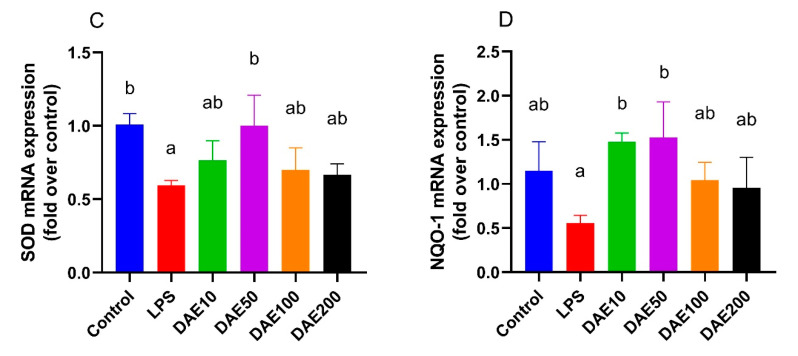
Effects of lipopolysaccharide (LPS) and dandelion aqueous extract (DAE) on the mRNA expression of hemeoxygenase 1 (*HO-1*) (**A**), cysteine uptake transporter (*XCT*) (**B**), superoxide dismutase (*SOD*) (**C**), and NADPH-quinone oxidoreductase 1 (*NQO-1*) (**D**) in MAC-T cells. Columns without a common lowercase letter (a, b, c) indicate significant difference among treatments (*p* < 0.05). LPS: 100 ng/mL LPS; DAE10: 100 ng/mL LPS and 10 μg/mL DAE; DAE50: 100 ng/mL LPS and 50 μg/mL DAE; DAE100: 100 ng/mL LPS and 100 μg/mL DAE; DAE200: 100 ng/mL LPS and 200 μg/mL DAE.

Antioxidative genes investigated in this study are down-stream genes regulated by the Nrf2 signal pathway. Treatments with 10 and 50 μg/mL DAE exhibited significantly higher (*p* < 0.05) cellular hemeoxygenase 1 (*HO-1*) and NADPH-quinone oxidoreductase 1 (*NQO-1*) mRNA expression than the LPS treatment (Figure 5A,D). Cells in the LPS group had lower (*p* < 0.05) cysteine uptake transporter (*XCT*) mRNA expression, whereas the supplementation of DAE at the levels of 10 and 50 μg/mL significantly improved (*p* < 0.05) the mRNA expression of the *XCT* gene compared with other groups (Figure 5B). The supplementation of DAE at the concentration of 50 μg/mL significantly improved (*p* < 0.05) mRNA expression of the *SOD* gene compared with LPS group, but did not differ from the control (Figure 5C)

## 3. Discussion

Cell apoptosis plays a crucial role in maintaining homeostasis in organisms with cell proliferation. However, excessive apoptosis stimulated by extracellular agents is reflected as increased cell death and reduced cell viability [21]. In the present study, the supplementation of DAE alleviated cell apoptosis induced by LPS and improved the cell viability of MAC-T cells. Nevertheless, a slightly decline of cell viability in the DAE100 and DAE200 groups may indicate the cytotoxic effects of DAE in relatively high concentration. This is consistent with a previous study focusing on a cytotoxicity assessment of dandelion extracts, which suggested that the cytotoxic effect may result from the content of flavonoid extracts, especially luteolin and luteolin 7-glucoside [11]. Moreover, the declined cell viability can be also explained by the inhibitory effects on cell proliferation due to relatively high polyphenol contents [22].

Normally, the production of ROS during oxygen metabolism and the elimination of ROS through antioxidant defenses maintain a dynamic equilibrium in organisms. However, over-production of ROS under certain conditions, such as pathogen infections, stresses, or abnormal metabolism, may result in oxidative stress [23]. In previous studies, increased production of ROS and oxidative damage markers were observed in MECs treated with LPS at the concentration of 100 ng/mL or higher, or in serum and tissue samples of dairy cows fed high-gram diets [3,24,25]. Our data confirmed the LPS induced oxidative stress in MAC-T cells by enhancing ROS production and cellular concentrations of MDA, 8-OHdG and PC, the oxidative damage markers of lipids, DNA, and proteins, respectively. In contrast, the production of ROS and oxidative markers was significantly decreased in the LPS-stimulated MAC-T cells after DAE treatments, especially in the 10 and 50 μg/mL DAE groups. To investigate the relationship between ROS scavenging and the antioxidant capability of DAE, we detected the activities of antioxidant enzymes. CAT and SOD are important in reducing the oxidative potential of intracellular superoxide radicals by catalyzing their catabolism into hydrogen peroxide and water, and GSH-Px degrades reactive oxygen intermediates to prevent them from interacting with critical cellular components, such as the phospholipids of bio-membranes, nucleic acids, and proteins [26,27]. Our results demonstrated that DAE, as an effective free radical scavenger, alleviated oxidative stress by relieving oxidative damage and promoting activities of antioxidant enzymes. However, the hyperactive effects of DAE on the antioxidants’ activities in MAC-T cells require further studies.

To further demonstrate the regulatory mechanism of DAE with respect to the Nrf2 pathway, we measured the protein expression of Nrf2 and Keap1, as well as the downstream gene expression of the Nrf2 signaling. Nrf2 is a critical transcription factor regulating the expression of antioxidant enzymes and protecting cells against oxidative-induced cytotoxicity [28,29]. Molecular and structural analyses of the Nrf2 pathway revealed a “dedepression” regulatory mechanism, wherein Nrf2 is anchored in the cytoplasm via Keap1-dependent ubiquitination-proteasomal degradation under normal physiological conditions in order to maintain antioxidant and cytoprotective enzymes at basal level and the cells in a stable state [30]. The Nrf2 pathway is stimulated by electrophiles and oxidants through the modification of critical cysteine thiols of Keap1 and/or phosphorylation of Nrf2, and the subsequently enhanced expression of antioxidant enzymes is achieved from the releasing of Nrf2 from Keap1 and translocating to the nucleus [31]. In the current study, protein expressions of both Nrf2 and Keap1 decreased after being treated with LPS, which was consistent with previous studies using different doses of pro-oxidants [7,20,32]. In contrast, the supplementation of DAE improved the expression of Nrf2 but declined expression of Keap1 at the DAE concentration of 50 μg/mL, which indicated that DAE induced the releasing of Nrf2 from Keap1.

Nrf2 directly regulates antioxidant defense systems via several mechanisms, including the induction of the catabolism of superoxide and peroxides, the regeneration of oxidized cofactors and proteins, the synthesis of reducing factors, increasing redox transport, and the induction of stress response proteins [30]. The downstream antioxidant genes of the Nrf2 signaling that were investigated in this study included *HO-1*, *XCT*, *SOD*, and *NQO-1*. As a stress response protein, HO-1 is the major enzyme with the ability to enhance antioxidant activity [33]. The redox transport, such as the cystine/glutamate transport, is supported by XCT, which has the capability to regulate cell defense in maintaining redox homeostasis [34]. NQO-1 is an important reducer in ARE-regulated drug metabolism and disposition [35]. In accordance with the results of Nrf2 and Keap1 protein expression, gene expressions of selected antioxidant enzymes were downregulated in the LPS treatment but upregulated after the supplementation of DAE at the concentration of 50 μg/mL in the present study. These findings were supported by previous studies focusing on the protecting effects of dandelion polyphenols against acetaminophen-induced hepatotoxicity in mice [36]. However, the trends of cellular gene expressions in the 10 μg/mL DAE group did not match up with those of Nrf2 and Keap1 protein expressions. This may result from other existing pathways or mediators that may activate the expressions of these genes, and this needs further study.

## 4. Conclusions

In conclusion, LPS at the concentration of 100 ng/mL induced oxidative stress through increasing ROS production and oxidative damage to cellular macromolecules and via inhibiting the mRNA expression of antioxidative genes. In contrast, the supplementation of DAE alleviated LPS-induced oxidative stress by activating the Nrf2 signaling pathway and upregulating its downstream gene expression.

## 5. Materials and Methods

### 5.1. Preparation of DAE

Dried dandelion flower and leaf were obtained from a hospital of traditional Chinese medicine (HeDaoTang, Chongqing, China) and identified by Dr. Zili Wang, a professor of traditional Chinese medicine at the Department of Veterinary Medicine, Southwest University, China. A voucher specimen (No. CAST201801) was deposited in the herbarium of Southwest University. The dried dandelion flower and leaf were extracted by following the procedures in a previous study [37]. Briefly, dried dandelion flower and leaf were grinded using an electric grinder, and 100 g powder was weighed and extracted three times with sterile water for 4 h at a temperature of 65 °C. The extract was filtered and then concentrated using a vacuum freeze dryer. The dried extract was dissolved in phosphate-buffered saline (PBS) at a concentration of 20 mg/mL. The contents of luteolin-7-O-glucoside and quercetin-7-O-glucoside in the DAE were measured using the high performance liquid chromatography method and following the protocol of the previous study [11], and the levels were 440.14 and 188.48 μg/g, respectively (Figure S1).

### 5.2. Cell Culture and Treatments

The MAC-T cells used in this study were kindly provided by Dr. Jianxin Liu and Dr. Hongyun Liu at the College of Animal Science, Zhejiang University, China. Cells were cultured in a CO_2_ incubator (Thermo Fisher Scientific, Waltham, MA, USA) at 37 °C, and culture medium was comprised of 90 mL of DMEM/F12 medium (Gibco, Grand Island, NY, USA), 10 mL of fetal bovine serum (Gibico, Grand Island, NY, USA), and 1 mL of penicillin-streptomycin solution (Hyclone, Logan, UT, USA).

MAC-T cells were firstly seeded into the culture medium without LPS and DAE, and after growing to 60% confluence the cells were divided into six groups or treatments (*n* = 6 per treatment) including the control, LPS (100 ng/mL LPS, Sigma, *Escherichia coli* O111:B4, Saint Louis, MO, USA), DAE10 (100 ng/mL LPS and 10 μg/mL DAE), DAE50 (100 ng/mL LPS and 50 μg/mL DAE), DAE100 (100 ng/mL LPS and 100 μg/mL DAE), and DAE200 (100 ng/mL LPS and 200 μg/mL DAE). MAC-T cells in each group were treated for 48 h, and culture medium was replaced every 24 h. The cells were collected after the treatment for further analysis.

### 5.3. Cell Viability Assay

Cell viability was detected using the cell counting kit-8 (CCK-8, Dojindo, Kumamoto, Japan). According to the manufacture’s protocol, 1 × 10^4^/mL cells were cultured in 96-well plates, and blanks with the same culture medium were set in wells without cells. In each well, 10 μL of CCK-8 reagent was added after 48 h treatment and incubated for another 2 h before measuring the optical density value at 450 nm using a microplate reader (Bio-Rad, xMark™, Hercules, CA, USA).

### 5.4. Reactive Oxygen Species Detection

The production of cellular ROS was measured with ROS detection kits (Jiancheng Bioengineering Institute, Nanjing, China) using the chemical fluorescence method. Based on the instruction of the manufacturer, 2 × 10^5^/mL MAC-T cells were incubated in 6-well plates for 48 h with different treatments. Then, culture medium was replaced with PBS containing 10 μM 2,7-dichlorofuorescin diacetate (DCFH-DA), and incubated for another 30 min. Cells were harvested in 500 mL PBS, and cellular fluorescence was measured with a flow cytometer (BD Biosciences, SanDiego, CA, USA) using the fluorescein isothiocyanate (FITC) detection method (excitation and emission wavelength at 488 and 525 nm, respectively). The data analysis was performed using the FlowJo^TM^ software V10.0 (Ashland, KY, USA).

### 5.5. Assay for Oxidative Damage Markers and Antioxidant Enzyme Activity

MAC-T cells were harvested after 48 h treatment, ultrasonically disrupted on ice, and centrifuged at 2500× *g* for 5 min at 4 °C. The supernatants were collected and stored at −20 °C for oxidative damage markers and antioxidant enzyme activity analyses. The contents of PC, 8-OHdG and MDA as well as the activities of SOD, CAT, GSH-Px, and T-AOC were determined using ELISA kits (Mlbio, Shanghai, China) by following the manufacturer’s instructions. Cellular protein concentrations were measured to normalize the above data using a bicinchoninic acid (BCA) protein assay kit (Sango Biotech, Shanghai, China).

### 5.6. RNA Isolation, cDNA Synthesis, and Quantitative Real-Time PCR (qPCR)

Total RNA of MAC-T cells was extracted using the TRIzol reagent (Invitrogen, Carlsbad, CA, USA) and following manufacturer’s instructions. The reverse transcription process was carried out using a cDNA synthesis kit (Bio-Rad, Hercules, CA, USA), and the incubation program consisted of 5 min at 25 °C, 20 min at 46 °C, and 1 min at 95 °C. qPCR reactions were conducted in the CFX96 Real-time system (Bio-Rad, Hercules, CA, USA) using the qPCR Kit (Bio-Rad, Hercules, CA, USA). The amplification and quantification were performed under the following procedures: pre-incubation for 30 sec at 95 °C, 40 cycles of denaturation for 5 sec at 95 °C, and annealing for 5 sec at 60 °C. Melting curves were analyzed to ensure the specific amplification, and the levels of relative mRNA expression were calculated using the 2-ΔΔCT method. Primers of antioxidant genes were designed using Primer Premier 5.0 software (Table 1), and synthesized by BGI Co., Ltd. (Shenzhen, China). Antioxidant genes include *HO-1*, *SOD*, *NQO-1*, and *XCT*. The glyceraldehyde-3-phosphate dehydrogenase gene (*GAPDH*) was used as a housekeeping gene.

### 5.7. Western Blot Analysis

Total protein of MAC-T cells was extracted using the Total Protein Extraction Kit (Sango Biotech, Shanghai, China) for the detection of Nrf2 and Keap1 protein expression. The BCA protein assay kit was used to measure total protein concentrations, and 20 μg protein was separated by SDS-PAGE (Genscript, Nanjing, China) at 140 V for 45 min and electrophoretically transferred to polyvinylidene fluoride membranes (GE Healthcare, Wasukesha, WI, USA). Membranes were blocked with 5% skim milk buffer for 1 h, and then incubated overnight at 4 °C with primary antibody against Nrf2, Keap1 or GAPDH (Proteintech, Wuhan, China). Subsequently, membranes were washed 3 times with Tris-HCl Tween buffer (TBST) and incubated at room temperature for 1 h with secondary antibody (HRP-conjugated Affinipure Goat Anti-Mouse IgG, Proteintech, Wuhan, China). Then membranes were washed 3 times with TBST, and then were developed with the Clarity Western ECL Substrate Reagent (Bio-Rad, Hercules, CA, USA). Finally, the membranes were visualized using the ChemiDocTM MP System (Bio-Rad, Herculers, CA, USA), and the band densities was measured using Image Lab 6.0.1 software (Bio-rad, Herculers, CA, USA).

### 5.8. Statistical Analysis

Data were expressed as the means ± standard error (SE), and statistical analysis was performed using SPSS 19.0 (IBM Inc., Armonk, NY, USA, 2010). One-way analysis of variance followed by Duncan’s multiple comparison was used to determine the statistical significance among variables of different treatments. Probability values (*p*-value) of less than 0.05 were considered statistically significant.

## Figures and Tables

**Table 1:** Primer sequences used for quantitative real-time PCR.

Gene	Primer Sequence	Product Size (bp)	GenBank Accession No.
*HO-1*	F: GGCAGCAAGGTGCAAGA	221	NM_001014912.1
	R: GAAGGAAGCCAGCCAAGAG		
*SOD*	F: GAGGCAAAGGGAGATACAGTC	197	NM_174615.2
	R: GTCACATTGCCCAGGTCTC		
*NQO-1*	F: GGTGCTCATAGGGGAGTTCG	235	NM_001034535.1
	R: GGGAGTGTGCCCAATGCTAT		
*XCT*	F: GATACAAACGCCCAGATATGC	136	XM_002694373.2
	R: ATGATGAAGCCAATCCCTGTA		
*GAPDH*	F: GGGTCATCATCTCTGCACCT	177	NM_001034034.2

*HO-1*: hemeoxygenase 1; *SOD*: superoxide dismutase; *NQO-1*: NADPH-quinone oxidoreductase 1; *XCT*: cysteine uptake transporter; *GAPDH*: glyceraldehyde-3-phosphate dehydrogenase.

## Supplementary Materials

The following are available online at https://www.mdpi.com/2072-6651/12/8/496/s1, Figure S1: The high performance liquid chromatography (HPLC) chromatograms for measuring the contents of luteolin-7-O-glucoside and quercetin-7-O-glucoside in dandelion aqueous extract (DAE).
