# Supplementary material for: Dandelion Extract Alleviated Lipopolysaccharide-Induced Oxidative Stress through the Nrf2 Pathway in Bovine Mammary Epithelial Cells
Source: Toxins (Basel). 2020 Aug 1;12(8):496. doi: 10.3390/toxins12080496 (PMC7472369; doi:10.3390/toxins12080496)
Supplement: Supplementary file 1 [file toxins-12-00496-s001.pdf]

Supplementary Materials: Dandelion Extract Alleviated Lipopolysaccharide-Induced Oxidative Stress through the Nrf2 Pathway in Bovine Mammary Epithelial Cells

Yawang Sun, Yongjiang Wu, Zili Wang, Juncai Chen, You Yang and Guozhong Dong

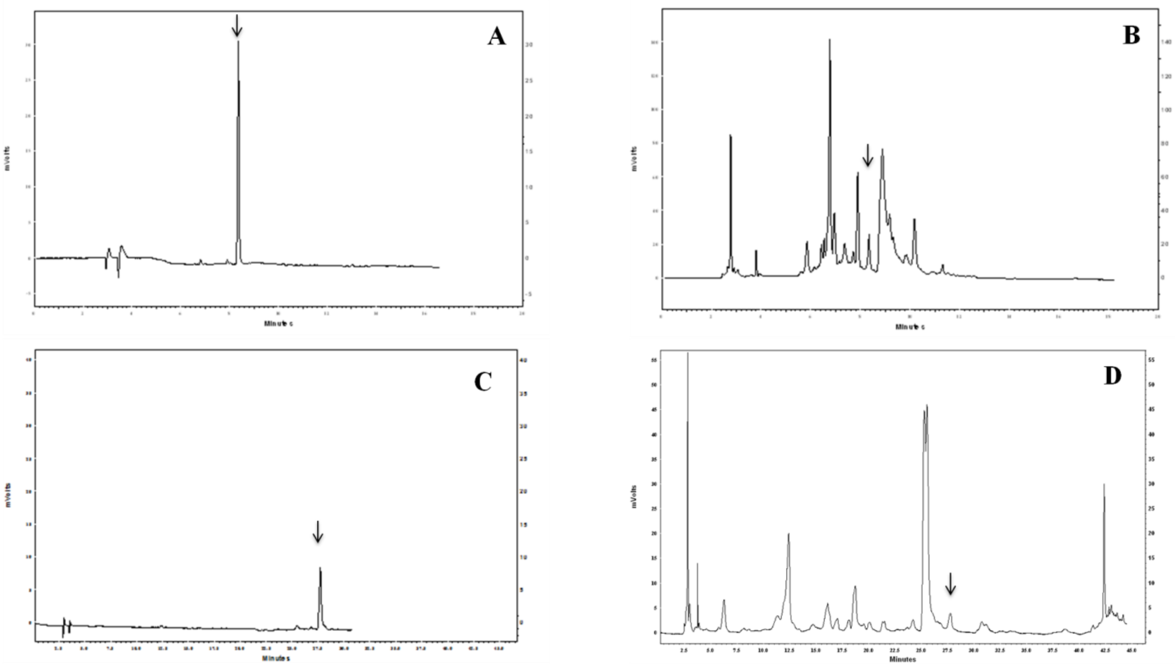

Figure S1. The high performance liquid chromatography (HPLC) chromatograms for measuring the contents of luteolin-7-O-glucoside and quercetin-7-O-glucoside in dandelion aqueous extract (DAE). (**A**) Standard luteolin-7-O-glucoside; (**B**) Luteolin-7-O-glucoside in DAE; (**C**) Standard quercetin-7-O-glucoside; (**D**) Quercetin-7-O-glucoside in DAE.
